# Chronic liver injury scores are superior prognostic indicators of outcomes in severe alcohol-related burns

**DOI:** 10.1038/s41598-025-07499-0

**Published:** 2025-06-25

**Authors:** Martynas Tamulevicius, Nadjib Dastagir, Khaled Dastagir, Peter M. Vogt, Florian Bucher

**Affiliations:** Department of Plastic, Aesthetic, Hand and Reconstructive Surgery, Hannover Medical School, Carl-Neuberg-Strasse 1, 30625 Hannover, Germany

**Keywords:** Chronic liver injury, Severe burns, Alcohol-related burns, Outcome prediction, Blood alcohol concentration, Prognostic markers, Liver diseases, Surgery

## Abstract

Acute and chronic alcohol abuse are common among burn patients and may be associated with chronic liver injury, a potential factor influencing outcomes. This study evaluates the predictive power of the blood alcohol concentration (BAC) and non-invasive liver fibrosis scores and their applicability in burn patients. A retrospective analysis was conducted on patients admitted to a high-volume supraregional burn center in Northern Germany between 2007 and 2024. Patients were categorized based on their BAC at admission: low (< 100 mg/dL) vs. high (≥ 100 mg/dL). Data collected included demographics, comorbidities, and outcomes. Non-invasive liver fibrosis markers such as the Fibrosis-4 (FIB-4) score, aspartate transaminase-to-platelet ratio index (APRI) and non-alcoholic fatty liver disease (NAFLD) fibrosis score were applied to both groups. Among 121 large-surface burn patients (mean total body surface area: 16.4%), no significant differences were observed between BAC groups in demographics, comorbidities, or ICU admission rates. The serum ethanol concentration showed no significant predictive value for mortality (AUC = 0.515). In contrast, the FIB-4 score (AUC = 0.781) and APRI (AUC = 0.736) demonstrated strong prognostic accuracy. In multivariate analysis, the Abbreviated Burn Severity Index (OR = 2.42; *p* = 0.001), serum albumin (OR = 0.29; *p* = 0.016), and FIB-4 score (OR = 1.50; *p* = 0.033) emerged as independent predictors of mortality. Propensity score matching analysis confirmed that BAC was not associated with increased mortality after adjustment for burn depth and extent. Non-invasive liver fibrosis markers, such as FIB-4 score, provide valuable prognostic insights in burn patients, independent of acute alcohol intoxication and should be considered a routine screening tool for large surface burn patients. Incorporating chronic liver dysfunction into existing burn severity models may enhance risk stratification and outcome prediction.

## Introduction

The World Health Organization attributes 2.6 million deaths annually to alcohol consumption, accounting for 4.7% of global deaths^1^. Additionally, alcohol consumption is a major risk factor for various liver and cardiovascular diseases, multiple types of cancer, and behavioral conditions such as depression and self-inflicted injury^2^. The global incidence of burn injuries is estimated at approximately 8 million cases per year, with up to 40% of adult patients presenting with an elevated blood alcohol concentration (BAC) at initial assessment^3,4^. This trend has been increasing over the years^5^. More than 1 in 10 of these patients meet the criteria for chronic alcohol abuse, defined as daily or near-daily consumption^6^. Both acute and chronic excessive alcohol consumption impair the healing process at multiple levels by weakening the immune response^7^, increasing susceptibility to infections^8^, and exacerbating oxidative stress due to a diminished hemodynamic counterregulatory response^9^. Chronic liver disease (CLD) is a major global health concern, affecting approximately 1.5 billion people^10^. Among its various forms, nonalcoholic fatty liver disease (NAFLD) is the most prevalent, accounting for 59% of cases, followed by hepatitis B virus at 29%, hepatitis C virus at 9%, and alcohol-related liver disease at 2%^11,12^. CLD often progresses silently, with gradual hepatic inflammation and fibrosis that can eventually lead to cirrhosis and an increased risk of primary liver cancer. Alcohol remains a significant contributor to liver disease, accounting for 30–50% of cirrhosis-related deaths worldwide^11^. Accurately assessing the prevalence of alcohol-related liver disease is challenging because diagnosis relies on self-reported alcohol consumption, which may be unreliable. By contrast, viral hepatitis can be objectively diagnosed through laboratory testing, making its burden easier to quantify. It is well known that cirrhosis significantly increases surgical risk, with perioperative mortality estimated to be three times higher in affected patients than in those without liver disease^13–15^.

Alcohol-related liver disease significantly impacts burn injury outcomes. Patients with preexisting liver disease have a higher mortality risk and longer hospital stays than patients without liver disease^16^. Alcohol may lower the threshold for postburn hepatic damage through various mechanisms, including modulation of extrahepatic events and alteration of the gut–liver axis^17^. The prevalence of alcohol-related burns is increasing^5^. Additionally, patients with liver disease are more likely to have comorbidities such as alcohol abuse, drug abuse, and psychiatric disorders^18,19^. These findings highlight the growing burden of alcohol on burn care and the need for targeted interventions.

Earlier studies have suggested a link between a positive BAC at admission and increased mortality in burn patients^20,21^. However, recent research by Bria et al.^22^ showed that while a higher BAC was associated with prolonged mechanical ventilation, it had no significant impact on mortality, length of hospital stay, or complications. These findings shift the focus away from acute alcohol intoxication and highlight the need to consider chronic liver injury (CLI), a well-established risk factor that may play a far more critical role in patient outcomes. Moreover, growing evidence suggests a strong connection between aging and CLI, with epidemiological data indicating a rising prevalence of liver fibrosis in older individuals. Studies have shown that aging is associated with progressive histopathological changes in the liver, including steatosis, central vein fibrosis, and perisinusoidal fibrosis^23,24^. Obed et al.^18^ highlighted the aging trend among burn patients, underscoring the need for further investigation into the impact of CLI on their clinical outcomes. Despite numerous pathophysiological studies emphasizing the detrimental effects of alcohol consumption on burn patients, widely used injury scoring systems for predicting burn mortality—such as the Abbreviated Burn Severity Index (ABSI), Revised Baux Score, FLAMES Score, and Burn Mortality Prediction Score—do not account for the BAC or the presence of CLD. This omission raises important questions about whether these factors should be integrated into existing models to improve risk assessment and outcome prediction in burn patients^25^.

The aim of this study was to evaluate the impact of the BAC at admission on burn patient outcomes and to assess the predictive value of widely used laboratory-based, non-invasive fibrosis scores at admission, including the aspartate transaminase (AST)-to-platelet ratio index (APRI), Fibrosis-4 (FIB-4) score, and NAFLD fibrosis score^26,27^. Given the growing recognition of CLD as a significant yet underappreciated factor in burn prognosis, we sought to determine whether these liver injury markers provide a more accurate outcome prediction than the BAC.

## Results

### Patient demographics and burn characteristics

During the study period, 1,018 patients with burn injuries were treated at our center. Of these, 121 (11.9%) met the inclusion criteria, including BAC testing at admission. Among them, 45 (37.2%) patients were classified into the BAC < 100 mg/dL group, and 76 (62.8%) patients were classified into the BAC ≥ 100 mg/dL group. Most patients were male (77.9%), with no significant difference between BAC groups (*p* = 1.000). The mean age of the cohort was 44.41 ± 15.45 years, with no statistically significant difference between the groups (*p* = 0.492). Similarly, the body mass index (BMI) was comparable between the groups (24.52 ± 4.73 vs. 25.44 ± 3.92 kg/m^2^, *p* = 0.273). The smoking prevalence was significantly higher in the BAC ≥ 100 mg/dL group (36.8%) than in the BAC < 100 mg/dL group (17.8%, *p* = 0.039). Although a history of alcohol abuse was more common in the BAC ≥ 100 mg/dL group (30.3%) than in the BAC < 100 mg/dL group (20.0%), this difference was not statistically significant (*p* = 0.287). The prevalence of diabetes and peripheral arterial disease was low across the cohort (3.3% and 4.1%, respectively), with no significant differences between groups (*p* = 0.628 and *p* = 0.156, respectively). Overall, 35.2% of patients had comorbidities, which were significantly more frequent in the BAC ≥ 100 mg/dL group (43.4%) than in the BAC < 100 mg/dL group (22.2%, *p* = 0.020).

The primary etiology of burns in the cohort was direct flame exposure (77.7%), followed by explosions (15.6%) and scalds (6.6%). There were no significant differences in burn etiology between the BAC groups (*p* > 0.617 for all comparisons). Deep partial-thickness burns were significantly more common in the BAC ≥ 100 mg/dL group (75.0%) than in the BAC < 100 mg/dL group (51.1%, *p* = 0.010). Similarly, full-thickness burns were more prevalent in the BAC ≥ 100 mg/dL group (46.1%) than in the BAC < 100 mg/dL group (26.7%, *p* = 0.034). The mean burned body surface area (BSA) was 16.36% ± 18.26%, with no significant difference between the BAC groups (17.53% ± 20.56% vs. 15.64% ± 16.80%, *p* = 0.600). The mean ABSI score was 5.6 ± 2.33 overall, with no significant differences between groups (*p* = 0.852). The patient and burn characteristics are summarized in Table 1.

**Table 1:** Patient demographics and characteristics.

*n* (%)	All (*n* = 121)	BAC < 100 mg/dl (*n* = 45)	BAC ≥ 100 mg/dl (*n* = 76)	*p* value
Gender (male)	95 (77.9%)	35 (77.8%)	60 (78.9%)	1.000
Age (y), mean ± SD	44.41 ± 15.45	43.13 ± 16.78	45.20 ± 14.64	0.492
BMI (kg/m²), mean ± SD	25.09 ± 4.25	24.52 ± 4.73	25.44 ± 3.92	0.273
Smoker	36 (29.5%)	8 (17.8%)	28 (36.8%)	**0.039**
Alcohol abuse	32 (26.2%)	9 (20.0%)	23 (30.3%)	0.287
Diabetes	4 (3.3%)	2 (4.4%)	2 (2,6%)	0.628
Peripheral arterial disease	5 (4.1%)	0 (0.0%)	5 (6.6%)	0.156
Comorbidities	43 (35.2%)	10 (22.2%)	33 (43.4%)	**0.020**
**Etiology**				
Flame	94 (77.69%)	34 (75.56%)	60 (78.95%)	1.000
Explosion	19 (15.6%)	8 (17.8%)	11 (14.5%)	0.617
Scalds	8 (6.6%)	3 (6.7%)	5 (6.6%)	1.000
Deep partial thickness burns	80 (65.6%)	23 (51.1%)	57 (75.0%)	**0.010**
Full thickness burns	47 (38.5%)	12 (26.7%)	35 (46.1%)	**0.034**
Burned body surface (%), mean ± SD	16.36 ± 18.26	17.53 ± 20.56	15.64 ± 16.80	0.600
ABSI, mean ± SD	5.6 ± 2.33	5.54 ± 2.45	5.63 ± 2.27	0.852
BAC level (mg/dl), mean ± SD	1.36 ± 0.74	0.63 ± 0.27	1.80 ± 0.55	**< 0.001**

Significant values are in bold.

## Admission characteristics

**Fig. 1:**
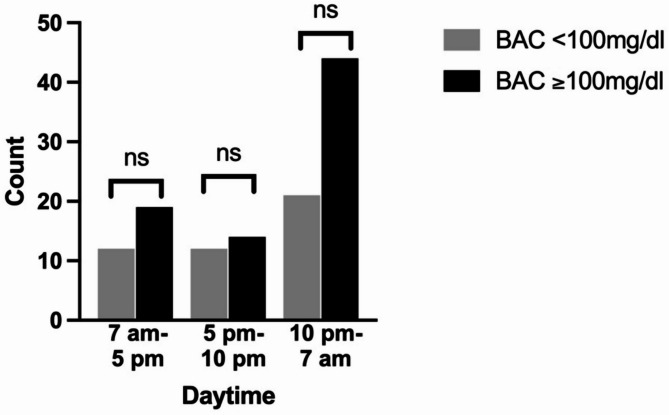
Seasonal distribution of admissions.

**Fig. 2:**
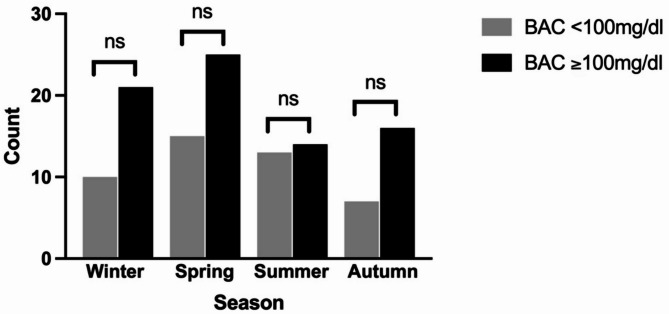
Distribution of admission timepoints during daytime.

The highest number of admissions for patients with BAC < 100 mg/dL occurred between June and August, while admissions for patients with BAC ≥ 100 mg/dL were most frequent from October to April. Although higher admissions of patients with elevated BAC levels were observed during the winter, spring, and autumn months, these differences were not statistically significant (*p* = 0.667, *p* = 0.960, and *p* = 0.632, respectively; see Fig. 1). Additionally, admissions predominantly occurred during the night shift, between 10 pm and 7 am, but this was also not statistically significant (*p* = 0.348; see Fig. 2).

## Outcomes

The mean length of hospital stay was 15.19 ± 21.13 days, with no significant difference between the BAC < 100 mg/dL group (13.04 ± 15.44 days) and the BAC ≥ 100 mg/dL group (16.46 ± 23.88 days; *p* = 0.342). Similarly, the mean number of intensive care unit (ICU) days did not differ significantly between the groups (*p* = 0.700). ICU admission was required for 81.1% of patients, with slightly higher rates in the BAC < 100 mg/dL group (86.7%) than in the BAC ≥ 100 mg/dL group (78.9%; *p* = 0.337). Intubation was required in 28.7% of patients, and inhalation injuries were observed in 27.9%, with no significant differences between the groups (*p* = 1.000 for both). Patients requiring at least one surgery were more frequent in the BAC ≥ 100 mg/dL group (52.6%) than in the BAC < 100 mg/dL group (46.7%), although the difference was not statistically significant (*p* = 0.575). The proportion of patients requiring three or more surgeries was slightly higher in the BAC ≥ 100 mg/dL group (45.0% vs. 38.1%; *p* = 0.786). Postoperative pneumonia occurred in 5.0% of patients, with no significant difference between the groups (6.7% in the BAC < 100 mg/dL group vs. 3.9% in the BAC ≥ 100 mg/dL group; *p* = 0.670). Wound infections were observed in 12.4% of patients, with no significant difference between the groups (11.1% in the BAC < 100 mg/dL group vs. 13.2% in the BAC ≥ 100 mg/dL group; *p* = 0.741). Mortality occurred in 14.0% of patients overall, with no significant difference between the BAC < 100 mg/dL group (11.1%) and the BAC ≥ 100 mg/dL group (15.8%; *p* = 0.593). The patient outcomes are summarized in Table 2.

**Table 2:** Surgical and functional outcomes.

*n* (%)	All (*n* = 121)	BAC < 100 mg/dl (*n* = 45)	BAC ≥ 100 mg/dl (*n* = 76)	*p* value
Length of stay, mean ± SD	15.19 ± 21.13	13.04 ± 15.44	16.46 ± 23.88	0.342
ICU days, mean ± SD	11.85 ± 20.39	11.00 ± 15.76	12.36 ± 22.78	0.700
Required ICU admission	99 (81.1%)	39 (86.7%)	60 (78.9%)	0.337
Required intubation	35 (28.7%)	13 (28.9%)	22 (28.9%)	1.000
Inhalation injury	34 (27.9%)	13 (28.9%)	21 (27.6%)	1.000
At least 1 surgery	61 (50.4%)	21 (46.7%)	40 (52.6%)	0.575
≥ 3 surgeries	26 (21.5%)	8 (38.1%)	18 (45.0%)	0.786
Postoperative pneumonia	6 (5.0%)	3 (6.7%)	3 (3.9%)	0.670
Wound infection	15 (12.4%)	5 (11.1%)	10 (13.2%)	0.741
Mortality	17 (14.0%)	5 (11.1%)	12 (15.8%)	0.593

To assess whether BAC independently predicted mortality after accounting for burn severity, we performed propensity score matching using burn size and the presence of 2b° and 3rd-degree burns as covariates. Patients with BAC ≥ 100 mg/dL were matched 1:1 to patients with BAC < 100 mg/dL, resulting in a matched cohort of 42 pairs (*n* = 84). After matching, the mortality rate was 14.3% in the BAC < 100 mg/dL group and 19.0% in the BAC ≥ 100 mg/dL group. After adjusting for burn depth and extent, no statistical significance was identified (*p* = 1.000).

## Analysis of outcomes using non-invasive laboratory scores for chronic liver injury (CLI)

**Fig. 3:**
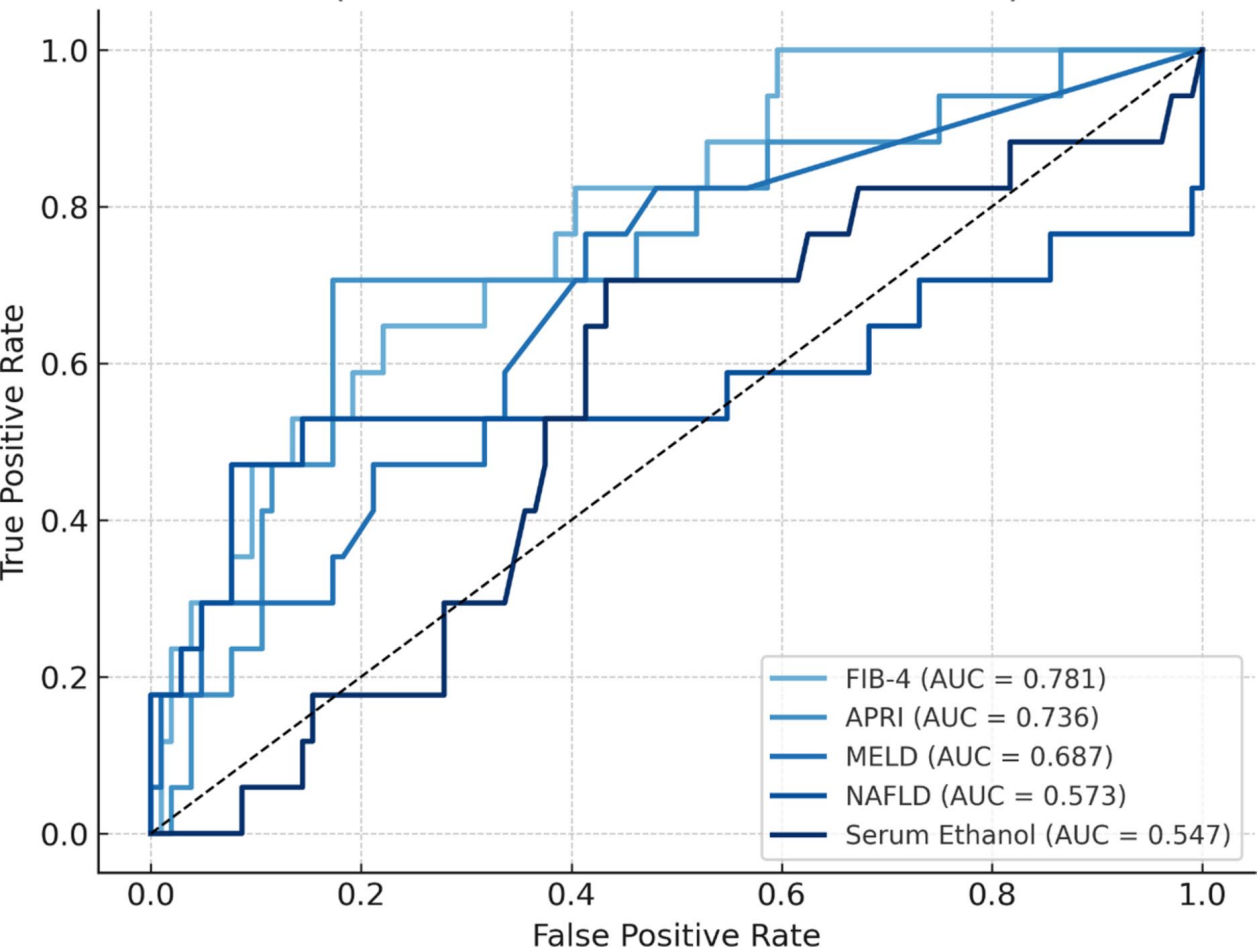
ROC curves for predicting mortality using laboratory scores for CLI and serum ethanol concentration.

The predictive ability of laboratory-based scores and serum ethanol concentration for mortality was assessed using the area under the receiver operating characteristic curve (AUC). The FIB-4 score demonstrated the strongest discriminatory ability, with an AUC of 0.781 (95% CI: 0.672–0.889, *p* < 0.001). Similarly, the AST-to-Platelet Ratio Index (APRI) showed good predictive performance, with an AUC of 0.736 (95% CI: 0.604–0.869, *p* < 0.001). In contrast, the MELD score and NAFLD fibrosis score exhibited limited predictive value, with AUCs of 0.687 (95% CI: 0.531–0.819, *p* = 0.010) and 0.573 (95% CI: 0.373–0.773, *p* = 0.475), respectively. Serum ethanol concentration showed no significant predictive capability, with an AUC of 0.547 (95% CI: 0.406–0.687, *p* = 0.515). These findings suggest that the FIB-4 score and APRI are useful for identifying patients at increased risk of mortality, whereas the MELD score, NAFLD fibrosis score, and serum ethanol concentration are less informative in this context (Fig. 3).

Based on the FIB-4 score, we analyzed the demographics and outcomes of patients according to their risk for CLI. Patients in the intermediate- to high-risk group were significantly older than those in the low-risk group (36.31 ± 13.16 vs. 54.82 ± 11.46 years, *p* < 0.001). BMI was comparable between the groups (24.98 ± 4.28 vs. 25.22 ± 4.37 kg/m^2^, *p* = 0.922), and serum ethanol levels also did not differ significantly (1.31 ± 0.69 vs. 1.42 ± 0.78 g/kg, *p* = 0.424). The mean burned BSA was higher in the intermediate- to high-risk group, although this difference was not statistically significant (14.15% ± 14.62% vs. 19.19% ± 21.88%, *p* = 0.153). However, the ABSI score was significantly higher in the intermediate- to high-risk group (4.93 ± 2.02 vs. 6.45 ± 2.44, *p* < 0.001), primarily because of the older age of these patients. No significant differences in burn etiology were observed between the groups. Patients in the intermediate- to high-risk group had significantly higher rates of smoking (20.6% vs. 41.5%, *p* = 0.016), alcohol abuse (17.6% vs. 37.7%, *p* = 0.021), peripheral arterial disease (0.0% vs. 9.4%, *p* = 0.014), and comorbidities (26.5% vs. 47.2%, *p* = 0.022). The prevalence of diabetes was similar across the groups.

In terms of outcomes, patients in the intermediate- to high-risk group had a longer mean hospital stay (12.75 ± 13.59 vs. 18.32 ± 27.83 days, *p* = 0.185) and ICU stay (10.21 ± 14.04 vs. 13.96 ± 26.41 days, *p* = 0.352), although neither difference was statistically significant. ICU admission, intubation, and inhalation injury rates were similar between the groups (*p* > 0.8 for all comparisons). However, the intermediate- to high-risk group had significantly more full-thickness burns (49.1% vs. 30.9%, *p* = 0.042) and required more extensive surgical interventions, with a higher rate of three or more surgeries (28.6% vs. 61.5%, *p* = 0.018). Postoperative complications were also more frequent in this group, including higher rates of wound infections (5.9% vs. 20.8%, *p* = 0.024). Mortality was significantly higher in the intermediate- to high-risk group (24.4%) than in the low-risk group (5.9%, *p* = 0.003). These findings suggest that patients with a higher FIB-4 score are at greater risk of severe outcomes, including mortality (Table 3).

**Table 3:** Outcomes based on risk of CLI according to FIB-4-Score.

*n* (%)	All (*n* = 121)	Low risk of CLI (*n* = 68)	Intermediate to high risk of CLI (*n* = 53)	*p* value
Length of stay, mean ± SD	15.19 ± 21.13	12.75 ± 13.59	18.32 ± 27.83	0.185
ICU days, mean ± SD	11.85 ± 20.39	10.21 ± 14.04	13.96 ± 26.41	0.352
Required ICU admission	99 (81.1%)	55 (80.9%)	44 (83.0%)	0.816
Required intubation	35 (28.7%)	19 (27.9%)	16 (30.2%)	0.841
Inhalation injury	34 (27.9%)	19 (27.9%)	15 (28.3%)	1.000
Deep partial-thickness burns	80 (65.6%)	45 (66.2%)	35 (66.0%)	0.987
Full-thickness burns	47 (38.5%)	21 (30.9%)	26 (49.1%)	**0.042**
At least 1 surgery	61 (50.4%)	35 (51.5%)	26 (49.1%)	0.855
≥ 3 surgeries	26 (21.5%)	10 (28.6%)	16 (61.5%)	**0.018**
Postoperative pneumonia	6 (5.0%)	3 (4.4%)	3 (5.7%)	1.000
Wound infection	15 (12.4%)	4 (5.9%)	11 (20.8%)	**0.024**
Mortality	17 (14.0%)	4 (5.9%)	13 (24.4%)	**0.003**

Significant values are in bold.

To account for potential confounding by age and burn size, two well-established predictors of mortality in burn patients, a propensity score matching analysis was performed using age and total body surface area (TBSA) as covariates. After 1:1 matching, 106 patients were included (53 per group). In the matched cohort, the mortality rate was 9.4% in the low FIB-4 group compared to 24.5% in the high FIB-4 group (*p* = 0.070). Although this result did not reach conventional statistical significance, the observed difference was clinically meaningful. A simulated power analysis demonstrated that statistical significance was achieved when the matched cohort was expanded to 1.5× its original size (*n* = 159), yielding a p-value of 0.022. To assess the impact of liver-related risk beyond mortality, we further evaluated secondary clinical outcomes within the matched cohort. No statistically significant differences were observed between FIB-4 risk groups for length of hospital stay (*p* = 0.436), ICU length of stay (*p* = 0.153), ICU admission (*p* = 0.786), intubation (*p* = 1.000), inhalation injury (*p* = 1.000), deep partial-thickness burns (*p* = 0.393), full-thickness burns (*p* = 1.000), wound infection (*p* = 1.000), postoperative pneumonia (*p* = 0.319), or surgical burden (≥ 1 surgery: *p* = 0.436; ≥3 surgeries: *p* = 0.680).

## Predictors of mortality: bivariate and multivariate analyses

The bivariate analysis identified several significant predictors of mortality (Table 4). Higher age (OR = 1.06, 95% CI: 1.02–1.11, *p* = 0.003), BMI (OR = 1.13, 95% CI: 1.01–1.27, *p* = 0.034), bilirubin (OR = 11.19, 95% CI: 2.53–49.38, *p* = 0.001), AST (OR = 1.02, 95% CI: 1.01–1.03, *p* < 0.001), and FIB-4 score (OR = 1.58, 95% CI: 1.22–2.06, *p* < 0.001) were associated with increased mortality. Additionally, mechanical ventilation, pneumonia, and wound infection significantly increased the risk. In the multivariate model, ABSI (OR = 2.42, 95% CI: 1.41–4.14, *p* = 0.001), albumin (OR = 0.29, 95% CI: 0.10–0.79, *p* = 0.016), and FIB-4 score (OR = 1.50, 95% CI: 1.03–2.17, *p* = 0.033) remained independent predictors of mortality. While diabetes showed a strong association in the bivariate analysis, it lost significance after adjustment. These findings highlight ABSI, albumin, and FIB-4 score as key independent risk factors for mortality.

**Table 4:** Bivariate and multivariate logistic regression analysis of cofactors for mortality.

Variable	Bivariate OR (95% CI)	*p*-value	Multivariate OR (95% CI)	*p*-value
Age at onset (years)	1.06 (1.02–1.11)	**0.003**	-	-
BMI (kg/m^2^)	1.13 (1.01–1.27)	**0.034**	-	-
Comorbidities (Yes vs. No)	1.75 (0.62–4.94)	0.288	-	-
Alcohol abuse (Yes vs. No)	1.64 (0.55–4.86)	0.376	-	-
Smoking (Yes vs. No)	0.69 (0.21–2.29)	0.547	-	-
Diabetes (Yes vs. No)	22.07 (2.15–227.08)	**0.009**	108.54 (0.00009–136,848.04)	0.513
Length of hospital stay (days)	0.99 (0.97–1.02)	0.681	-	-
Serum ethanol (g/kg)	1.14 (0.57–2.27)	0.717	-	-
Platelet count	1.01 (1.00–1.02)	**0.002**	-	-
INR	2.00 (0.37–10.87)	0.422	-	-
Quick-Test	1.00 (0.99–1.01)	0.934	-	-
Sodium (mmol/L)	1.04 (0.94–1.15)	0.428	-	-
Albumin (g/dL)	0.24 (0.10–0.57)	**0.001**	0.29 (0.10–0.79)	**0.016**
Bilirubin (mg/dL)	11.19 (2.53–49.38)	**0.001**	2.60 (0.37–18.11)	0.335
AST (GOT)	1.02 (1.01–1.03)	**< 0.001**	-	-
FIB-4 score	1.58 (1.22–2.06)	**< 0.001**	1.50 (1.03–2.17)	**0.033**
ICU stay (days)	1.00 (0.98–1.03)	0.714	-	-
Mechanical ventilation (Yes vs. No)	4.51 (1.56–13.10)	**0.006**	0.91 (0.18–4.67)	0.913
Surgery count	1.23 (1.00–1.52)	**0.050**	-	-
Pneumonia (Yes vs. No)	7.21 (1.32–39.30)	**0.022**	3.57 (0.19–66.15)	0.392
Wound infection (Yes vs. No)	3.92 (1.15–13.40)	**0.030**	0.30 (0.04–2.05)	0.221
ABSI	2.585 (1.71–3.92)	**< 0.001**	2.42 (1.41–4.14)	**0.001**

Significant values are in bold.

**Fig. 4:**
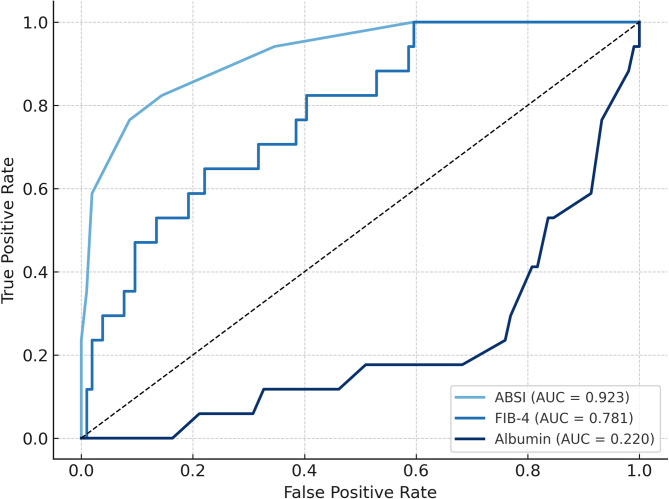
ROC curves for predicting mortality using ABSI, FIB-4 score and serum albumin concentration.

The receiver operating characteristic analysis (ROC) demonstrated that ABSI (AUC = 0.923) had the highest predictive accuracy for mortality, confirming its strong role in burn prognosis (Fig. 4). The FIB-4 score (AUC = 0.781) showed good discriminatory ability, reinforcing the relevance of CLI as an independent prognostic factor. By contrast, albumin (AUC = 0.220) had poor predictive value. Additionally, regression analysis demonstrated that while ABSI remained the strongest independent predictor of mortality (OR = 2.585, *p* < 0.001), incorporating FIB-4 improved the overall model fit. The combined model (ABSI + FIB-4) significantly outperformed ABSI alone (likelihood ratio test: χ^2^ = 4.004, *p* = 0.045), suggesting that CLI provides additional prognostic value. However, when adjusted for ABSI, FIB-4’s effect size decreased and became borderline significant (OR = 1.455, *p* = 0.056), indicating that its predictive role is secondary to burn severity. These findings suggest that integrating CLI markers into existing burn mortality models may enhance risk stratification, particularly in patients with preexisting liver dysfunction.

## Discussion

In this study, we analyzed the impact of alcohol consumption prior to burn injury on overall outcomes and quantified the predictive power of laboratory scores for CLI and the serum ethanol concentration. Large cohort studies have found no association between the BAC and mortality in burn patients^22,28^. However, elevated serum alcohol concentrations have been linked to prolonged hospital stays^29^. Similar findings were observed in our cohort, as patients with a high BAC had a longer mean hospital stay of 16.46 days and required ICU admission in 86.7% of cases. These results align with those reported by Holmes et al.^30^ and Griffin et al.^31^. Although patients in the high BAC cohort had significantly more deep partial- and full-thickness burns, as well as more comorbidities, BAC levels did not correlate with surgical or functional outcomes, including postoperative complications and mortality. Similar findings regarding the impact of BAC levels at admission on outcomes have been reported by other authors^22^. The prolonged hospital stay in patients with a high BAC may be attributed to increased initial medical needs rather than poorer recovery. These results suggest that BAC levels alone may not be the primary determinant of worse outcomes. Instead, the chronicity of alcohol use might play a more significant role because CLI is a well-established factor contributing to poorer outcomes in burn patients^16^.

Previous studies have investigated seasonal trends in burn center admissions and injury mechanisms, with varying results. Some studies have reported admission peaks during the summer and spring months^32,33^, while others have observed higher rates of thermal injuries during the winter period^34^. In our cohort, admissions with a low BAC peaked between June and August, whereas admissions with a high BAC were most frequent from October to April. Most admissions occurred during the night shift (10:00 pm to 7:00 am), with no statistically significant difference between BAC groups. The primary mechanism of injury was direct thermal injury caused by flames. These seasonal differences may be attributed to variations in burn injury mechanisms; for example, winter burns may be more often related to heating sources, whereas summer burns are more likely linked to outdoor activities. However, a detailed analysis of these patterns was not the focus of this study.

Liver function plays a crucial role in the body’s healing process after thermal injuries^16^. Severe trauma, such as burn injuries, triggers a shift in hepatic protein synthesis from constitutive proteins like albumin, transferrin, and retinol-binding protein to acute-phase proteins, which mediate the inflammatory response^35^. Advanced stages of liver fibrosis or cirrhosis are known to negatively impact surgical outcomes in burn patients^36^. Additionally, burn patients with preexisting liver disease have a higher mortality risk and longer hospital stays^16^, and elevated liver fibrosis markers have been associated with increased postoperative mortality following surgical intervention^37^. This raises the question of whether these parameters can be utilized to predict outcomes in burn patients. One objective of this study was to evaluate the predictive value of non-invasive indicators of liver fibrosis in patients with elevated serum alcohol levels. Chronic liver damage is highly suspected in patients with a BAC of > 100 mg/dL at the admission. The FIB-4 score and APRI have demonstrated excellent non-invasive prognostic power in patients with hepatocellular carcinoma^38,39^. Similar findings were observed in burn patients, with strong predictive ability for both the FIB-4 score (AUC = 0.781) and APRI (AUC = 0.736) in outcome prediction. Based on the FIB-4 score, patients in the intermediate- to high-risk group had a longer mean hospital and ICU stay, although without a statistically significant correlation. However, this group had significantly more full-thickness burns (*p* = 0.042), required more surgical interventions (*p* = 0.018), and had significantly higher rates of postoperative wound infections (*p* = 0.024), leading to a significantly higher mortality rate (*p* = 0.003). The multivariate analysis identified the ABSI score, serum albumin, and FIB-4 score as key independent risk factors for mortality following burn injury. In order to remove well-established predictors of mortality such as age and burn size as confounders, we performed propensity score matching on these variables. This allowed us to better isolate the prognostic value of the FIB-4 score. After matching, mortality and secondary outcome differences between FIB-4 risk groups remained clinically relevant, suggesting an independent effect of liver-related risk. Although statistical significance was not reached in the original matched cohort (*p* = 0.070), the effect size was considerable. A simulated power analysis showed that significance was achieved once the cohort exceeded 1.5× its original size, with increasing significance at larger scales. This supports the interpretation that the non-significant finding was likely due to limited statistical power. Moreover, when analyzing other outcomes, such as length of hospital stay, ICU stay, inhalation injury, burn depth, wound infection, postoperative pneumonia, and surgical burden, no statistically significant differences were observed. These results suggest that while a high FIB-4 score may be associated with a clinically relevant increase in mortality, it does not appear to predict other major clinical outcomes in this matched population.

Interestingly, the serum ethanol concentration did not demonstrate significant predictive capability (AUC = 0.515), a finding consistent with previous studies^22,40^. This supports the idea that the BAC alone is insufficient for assessing liver function and highlights why fibrosis markers provide a more accurate prognosis. To further explore this, we performed a propensity score matching analysis to assess whether BAC independently predicted mortality after accounting for burn severity. Matching was conducted using burn size and the presence of 2b° and 3rd-degree burns as covariates. Although a numerical difference was observed (14.3% vs. 19.9%), it did not reach statistical significance, suggesting that elevated BAC at admission is not a strong independent predictor of mortality once burn depth and extent are considered.

Popular injury scoring systems for predicting mortality following burn injury do not account for the serum BAC or liver function. Based on our findings, we strongly recommend modifying these scoring systems to incorporate these factors. Our results suggest that non-invasive liver fibrosis markers, such as FIB-4 and APRI, may serve as valuable tools for identifying burn patients at higher risk of adverse outcomes. Among these, the FIB-4 score demonstrated the highest discriminatory ability for predicting mortality. Based on these findings, FIB-4 screening could be integrated into the standard admission protocol for patients admitted to the burn intensive care unit. The score is derived from routinely collected laboratory parameters (age, AST, ALT, and platelet count) and can be calculated rapidly using validated online tools, making it a highly feasible and pragmatic option for clinical implementation. Integrating these scores into clinical decision-making could facilitate early risk stratification and tailored treatment approaches, particularly in patients with high BAC levels in whom CLD is suspected. Burn centers often face resource shortages, making effective triage essential; however, current referral guidelines do not consider the serum BAC or liver function^41^. Under existing criteria, patients with a total BSA of < 10% would not qualify for referral to a burn center regardless of liver function, potentially leading to adverse outcomes. These findings are particularly relevant because both acute and chronic alcohol abuse are more prevalent among burn patients, especially in flame-related injuries such as residential fires^42,43^—a pattern also reflected in our cohort. The strong predictive power of liver function markers in mortality risk assessment is further emphasized by the fact that all laboratory values used for calculation were obtained at admission, allowing for early and precise risk stratification. Given these findings, high-risk patients could be identified immediately upon admission, enabling targeted intervention. Current research suggests that liver fibrosis is potentially reversible if the underlying cause is addressed^43^; therefore, patient education following acute-phase stabilization could be a key component of long-term recovery. Additionally, alcohol withdrawal management should be incorporated into rehabilitation because these patients tend to have significantly higher inpatient rehabilitation rates and are at risk of poorer functional outcomes^40^.

This study had several limitations. First, because this was a single-center retrospective study, the generalizability of our findings may be limited. A multicenter approach would be necessary to validate these results across diverse patient populations. Second, the BAC levels may have been underreported because testing was performed at the discretion of the attending physician rather than through a standardized protocol, potentially introducing selection bias. This may have been particularly problematic in patients who were secondarily transferred from other facilities where BAC levels were not recorded upon initial admission. The supraregional burn center covers an area of approximately 50,000 km². Depending on transportation modality and duration, BAC levels may decline before arrival at the burn intensive care unit. A back-calculation of BAC was not performed, as patients were typically ventilated and sedated, leaving them unable to provide information about the timing of their last alcohol intake. Third, length of hospital stay (LOS) was reported as the total inpatient duration, and included patients who died during hospitalization. LOS was not normalized for mortality, which may have influenced the reported mean values, as deceased patients typically have shorter stays. Fourth, the relatively small sample size (121 of 1,018 patients treated during the study period) may have limited statistical power, particularly in the matched-pair analyses, although it was still sufficient to allow for meaningful clinical interpretation. Finally, only non-invasive indicators of CLI were used, as invasive diagnostic tools such as liver biopsy were not feasible in this vulnerable patient population. However, the APRI and, in particular, the FIB-4 score are well-established surrogate markers for chronic liver disease (CLD) and provide clinically relevant insights based on routine admission laboratory values. Future research should adopt a prospective, multicenter design with standardized protocols for BAC testing, liver function assessment, and extended liver diagnostics to further evaluate the role of CLD in burn patient outcomes.

Our study is the first to demonstrate that non-invasive liver fibrosis markers can predict mortality in burn patients with an elevated BAC. The multivariate analysis identified ABSI, serum albumin, and FIB-4 as independent risk factors, whereas BAC alone showed no predictive value. Notably, integrating FIB-4 into ABSI improved the predictive model, suggesting that CLD compounds the burn severity risk. These findings highlight the need to shift the focus from acute intoxication to underlying liver dysfunction when assessing the burn prognosis. Future research should aim to validate these results in larger, multicenter studies with standardized protocols for liver function assessment and burn severity scoring. Additionally, further investigation is needed to determine whether incorporating FIB-4 into clinical decision-making could enhance outcome prediction and improve risk stratification in burn patients.

## Methods

### Study design

In this retrospective, cross-sectional study, patients were referred to our European Burns Association-certified supraregional burn center, which provides specialized care beyond a single region, serving patients at both national and international levels. Admissions occurred via ambulance or helicopter, either as primary presentations or secondary transfers, between January 2007 and December 2024. Data for all patients admitted to and treated in the department’s burn care unit were collected anonymously through an online portal. Patients or their legal representatives provided informed consent for the use of their anonymized data. This retrospective study was approved by the local ethics committee of Hannover Medical School under the registration number 11844-BO-K-2025.

The aim of this study was to evaluate the impact of a positive blood alcohol concentration (BAC) at admission on outcomes in burn patients and to assess the predictive performance of non-invasive liver fibrosis scores. Patients with a positive BAC were included because this subgroup frequently exhibits chronic alcohol use, which is known to predispose individuals to chronic liver injury and fibrosis. Given the high heterogeneity of burn patients with respect to body composition, nutritional status, and comorbidities, restricting the cohort to BAC-positive individuals allowed for a more clinically uniform study population and reduced potential confounding. BAC was measured via serum ethanol concentration on admission as part of routine clinical care. The inclusion criteria were an age of ≥ 18 years, the presence of a burn injury requiring inpatient treatment, and a positive BAC at admission. The exclusion criteria were: minor burns not requiring hospitalization, age under 18 years, a negative blood alcohol concentration (BAC) level, incomplete medical records, missing follow-up data, severe comorbidities that could independently affect outcomes, end-stage liver disease (e.g., cirrhosis with decompensation) present at admission, and active malignancies. Severe comorbidities included previous organ transplantation requiring immunosuppressive therapy, active autoimmune diseases such as rheumatoid arthritis under systemic immunosuppression, and untreated HIV infection. These conditions were excluded to reduce confounding, as they are known to significantly influence immune response, wound healing, and mortality independent of burn severity.

**Fig. 5:**
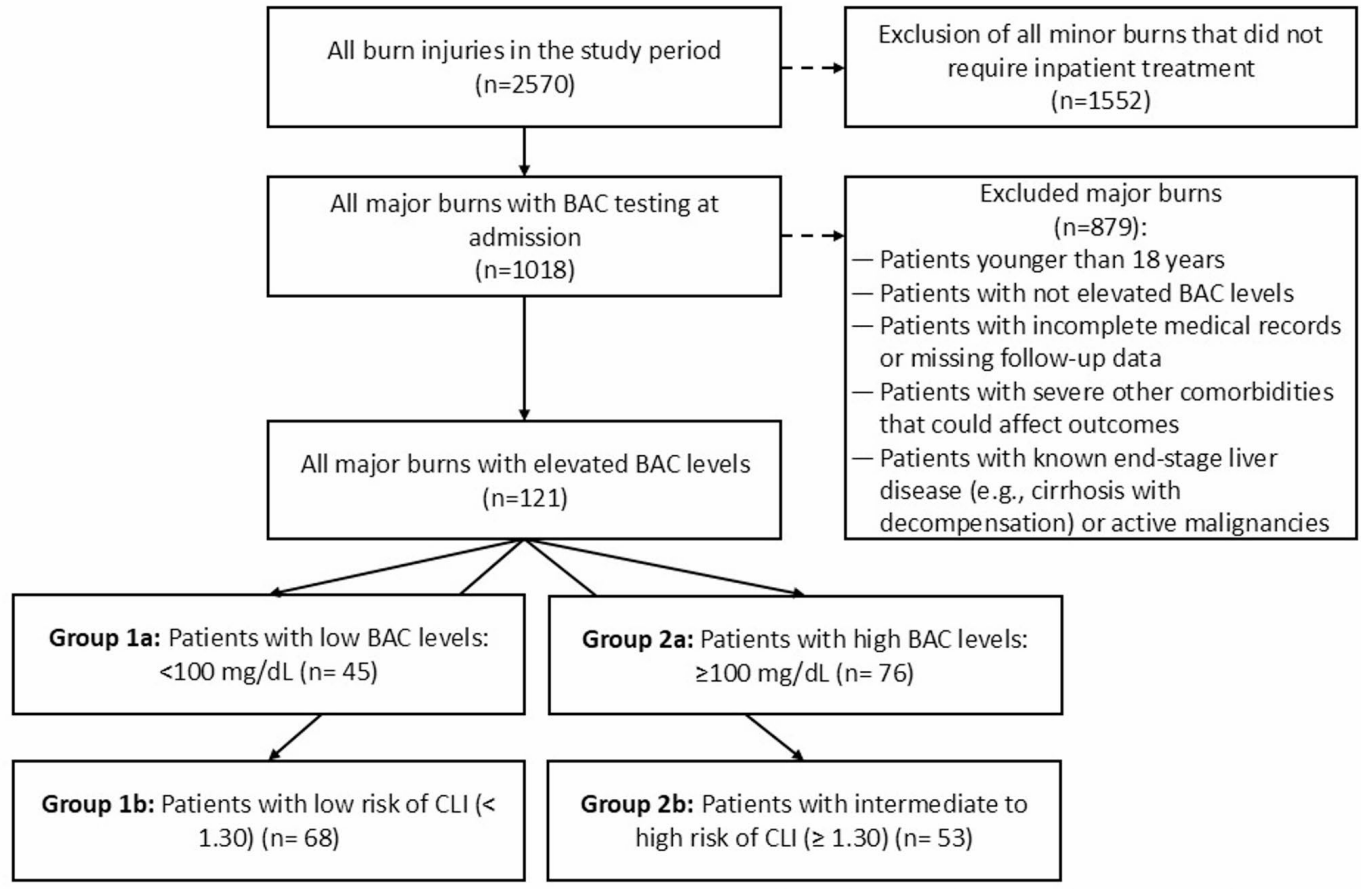
Flowchart of the study.

**Fig. 6:**
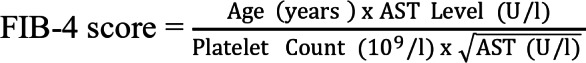
Formula for FIB-4 score.

The patients were categorized into two groups based on their BAC: low (< 100 mg/dL) and high (≥ 100 mg/dL) (Fig. 1). BAC levels were measured upon arrival at the emergency department at the discretion of the attending physician but were not routinely included in burn assessments. Additionally, we used the FIB-4 score, APRI and NAFLD fibrosis score, each of which is based on laboratory values at admission, for the noninvasive assessment of liver fibrosis as markers of chronic liver damage and potential indicators of CLD (Fig. 5). To evaluate the impact of CLI on outcomes, the patients were further stratified into two groups according to their FIB-4 score: low risk of CLI (< 1.30) and intermediate to high risk of CLI (≥ 1.30) (Fig. 6). This classification allowed for a comparative analysis of burn severity and outcomes based on underlying liver dysfunction, independent of acute alcohol intoxication.

### Propensity score matching

To minimize confounding and improve the validity of between-group comparisons, we performed propensity score matching (PSM) using a logistic regression model to estimate the likelihood of group assignment based on relevant baseline characteristics. Matching was conducted in a 1:1 nearest neighbor fashion without replacement, using a ranking algorithm to identify the best available matches and minimize distance in propensity scores. This approach prioritized covariate similarity and overall match quality. Covariate balance was assessed using descriptive statistics and standardized mean differences (SMDs), with all matched variables demonstrating good balance (SMDs < 0.1). The resulting matched cohort was used for all adjusted outcome analyses.

### Estimation of sample size

Based on a Type I error (α) of 0.05, a power (1-β) of 0.80, and an assumed medium effect size (Cohen’s d = 0.5), the estimated minimum sample size required for this study was approximately 64 patients per group (128 total) to achieve sufficient statistical power for detecting differences in outcomes between the BAC groups.

### Statistical analysis

Statistical analyses were performed using GraphPad Prism 10.3.1 (GraphPad Software, Inc., La Jolla, CA, USA), Microsoft Excel 16.78 (Microsoft Corp., Redmond, WA, USA), and IBM SPSS 30.0 (IBM Corp., Armonk, NY, USA). Descriptive statistics are presented as numbers (percentages) for categorical variables and medians with interquartile ranges for continuous variables. A p-value of < 0.05 was considered statistically significant. For categorical variables, Pearson’s chi-squared test was used, with Fisher’s exact test applied when > 20% of expected frequencies were < 5. For comparisons involving multiple groups, the Fisher–Freeman–Halton exact test was used. Normally distributed continuous variables were analyzed using the unpaired t-test for two groups and one-way ANOVA with Bonferroni correction for multiple groups. Non-normally distributed continuous variables were compared using the Mann–Whitney U test for two groups and the Kruskal–Wallis H test for multiple groups. Correlations between continuous variables were assessed using Pearson’s correlation for normally distributed data and Spearman’s correlation for non-normally distributed data.

To identify independent predictors of mortality and burn outcomes, a multivariate logistic regression analysis was performed, adjusting for total BSA burned, inhalation injury, age, sex, BMI, preexisting comorbidities (e.g., diabetes, cardiovascular disease), laboratory values, and CLD markers (FIB-4, APRI, and NAFLD fibrosis score). Variables included in the multivariate analysis were selected based on their clinical relevance and statistical significance in the univariate analyses (*p* < 0.10). Collinearity was assessed using variance inflation factor (VIF) analysis, with variables having a VIF of > 5 considered highly collinear. Consequently, the FIB-4 score and ABSI score were analyzed separately in independent models due to their high VIF values. A stepwise logistic regression model was used to determine the most significant variables associated with mortality.

## Data Availability

The data supporting the findings of this study are available from the corresponding author upon reasonable request. Due to privacy concerns, the dataset is not publicly archived. Access to the data will be provided in compliance with institutional guidelines and applicable regulations to ensure individual privacy is protected.
